# Identification of the mandibular landmarks in a pediatric population

**DOI:** 10.4317/medoral.18980

**Published:** 2013-10-13

**Authors:** Kenan Cantekin, Ahmet E. Sekerci, Ozkan Miloglu, Suleyman K. Buyuk

**Affiliations:** 1 DDS, PhD, Assistant Professor, Department of Paediatric Dentistry, Faculty of Dentistry, Erciyes University, Kayseri, Turkey; 2 DDS, PhD, Assistant Professor, Department of Maxillofacial Radiology, Faculty of Dentistry, Erciyes University, Kayseri, Turkey; 3 DDS, PhD, Assistant Professor, Department of Maxillofacial Radiology, Faculty of Dentistry, Ataturk University, Erzurum, Turkey; 4 DDS, Research Assistant, Department of Orthodontics, Faculty of Dentistry, Erciyes University, Kayseri, Turkey

## Abstract

Objectives: The aim of this study was to determine and compare the reliability to accomplish of common mandibular landmarks and to determine the incidence of incisive canals, anterior looping, and lingual foramina in children from panoramic and CBCT images. Study Design: Panoramic and CBCT images from 100 children and adolescent patients were randomly selected. In order to grade the visibility of mandibular anatomical landmarks, a four-point rating scale was used. Results: In panoramic images, the mandibular canal could be observed in 92.5% of cases, with good visibility in 12.0%. The mental foramen could be observed in 44.5% of cases, while none had good visibility. Anterior looping of the mental nerve was present in 16.5% of the cases, and none had good visibility. An incisive canal could be identified in 22.5% of cases, with only 1.5% showing good visibility. The lingual foramen could be visualized in 61.0% of cases, with good visibility in 6%. In CBCT images, the mandibular canal, the mental foramen, and the lingual foramen could be observed in 100% of the cases, with good visibility in 51.0%, 98.5%, and 45.0% of cases, respectively. Anterior looping of the mental nerve was present in 26% of cases, with 2% having good visibility. An incisive canal could be identified in 49.5% of cases, with only 75% showing good visibility. Conclusions: This study confirms the applicability of CBCT images to visualize critical structures in children.

** Key words:**Panoramic radiography, cone beam computed tomography, anatomical landmark.

## Introduction

Today, panoramic radiography is often used for primary evaluations in dental practice to obtain information about the teeth, upper and lower jawbones, sinuses, temporomandibular joints, and other hard tissues of the head and neck. However, panoramic radiographies only give two-dimensional information regarding the superimposition of all structures and lack information in a bucco-lingual direction. Contemporary imaging techniques such as cone beam-computed tomography (CBCT) may be particularly suitable in the evaluation of jaws, as three-dimensional visualization and the high-resolution analysis of the entire mandible provide adequate information to localize anatomical structures (1-3).

So far, several reports have been presented to locate and measure mandibular anatomical landmarks in adults using different radiological techniques, as visualized on panoramic (4-7) or CBCT images (3,8,9). However, no studies have been done to determine and compare the visibility of mandibular landmarks in children. The aim of this study was to determine the reliability of using panoramic and CBCT images to identify and compare common mandibular landmarks and to determine the incidence of incisive canals, anterior looping, and lingual foramina in children.

## Material and Methods

We defined five landmarks and collected data directly from mandibles and indirectly from panoramic and CBCT images of the mandibles in the same children. The anatomical landmarks in mandibles were the following: (1) the mandibular canal, (2) the mental foramen, (3) the anterior looping of the mental nerve, (4) the incisive canal, and (5) the lingual foramen.

Panoramic and CBCT images from 100 children and adolescent patients were randomly selected from existing records in the Department of Oral and Maxillofacial Radiology at the University of Erciyes, Kayseri, Turkey. All of the patients had been referred for CBCT diagnosis and treatment planning, consisting of 23 impacted teeth patients, 47 orthodontic patients, 19 possible pathosis patients, five supernumerary teeth patients, and six TMJ disorder patients ( Table 1).

**Table 1:**
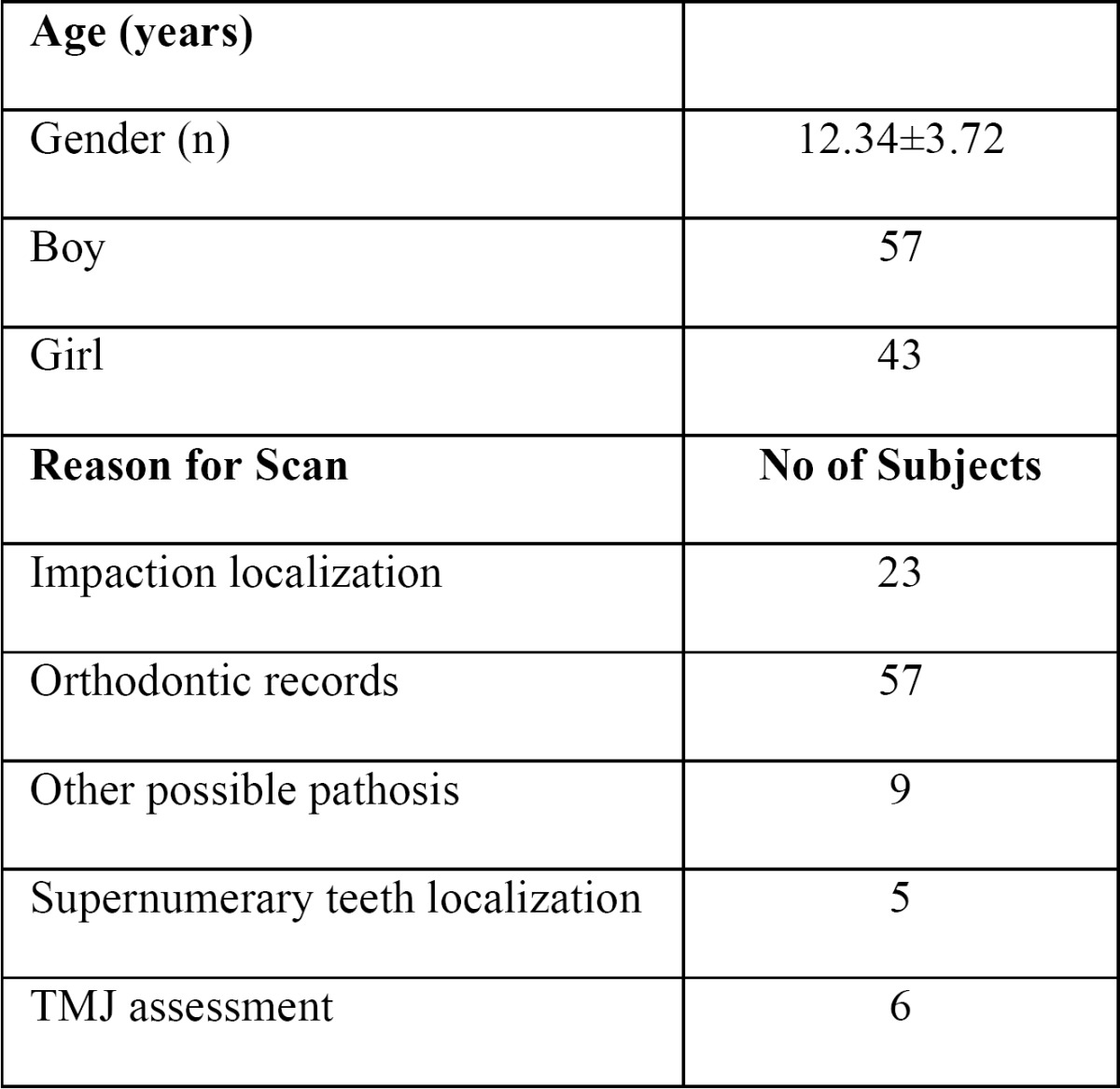
Description of the subjects and their indications for cone beam CT (CBCT).

All radiographs were performed by x-ray technicians who had a minimum of five years of work experience as of 1996, using an orthpantomography device (Planmeca Proline CC 2002, 60–80 kVp, 8–10 mA, 12.8 second exposure time, Helsinki, Finland) with a magnification factor of 1.2. The cone beam images were acquired using a Newton 5G (Quantitative Radiology, Verona, Italy) Flat panel-based CBCT machine. To establish a consistent orientation in the images, each patient was placed in a horizontal position such that the Frankfort horizontal plane (the plane between the highest point of the external auditory canal’s opening and the orbit’s lowest point) was perpendicular to the table, with the head within the circular gantry housing the x-ray tube. The x-ray tube detector system performed a 360° rotation around each patient’s head, with a scanning time of 36 s. The scanner operated with a maximum output of 110 KV and 15 mAs, a 0.16-mm voxel size and a typical exposure time of 5.4 s. The QR-NNT software version 2.21 (Quantitative Radiology) was used to analyze the images. Approval from the ethics committee was not required for this retrospective study.

In order to grade the visibility of mandibular anatomical landmarks, the following four-point rating scale was used.

No visibility = important structures are not visualized

Poor = important structures are not diagnostic

Moderate = important structures are diagnostic but could be improved

Good = important structures are optimally visualized

**Figure 1:**
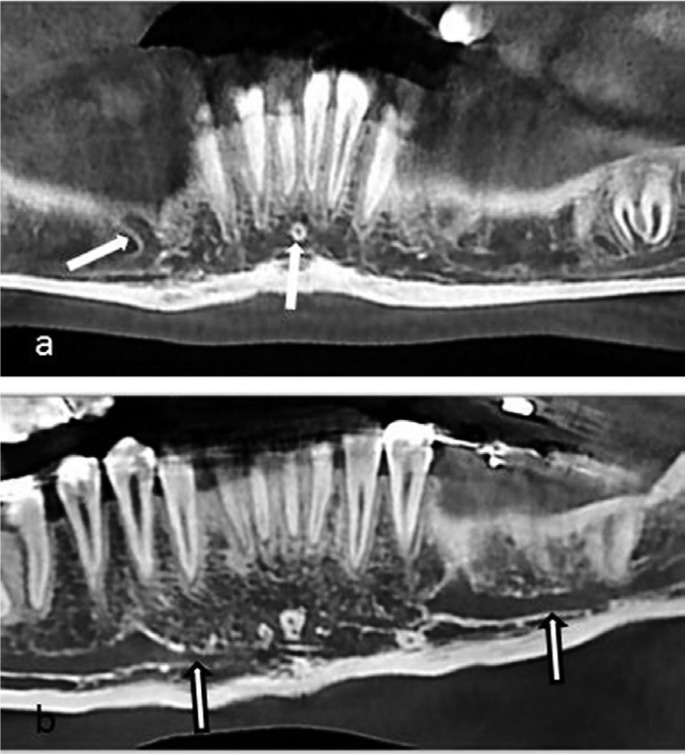
Cropped cone beam computed tomographic panoramic view of a 17-year-old boy with denoted structures; anterior looping of the mental nerve (right side) and lingual foramen (a), and incisive canal (right side) and mandibular canal (left side) of a 16-year old a girl (b).

**Figure 2:**
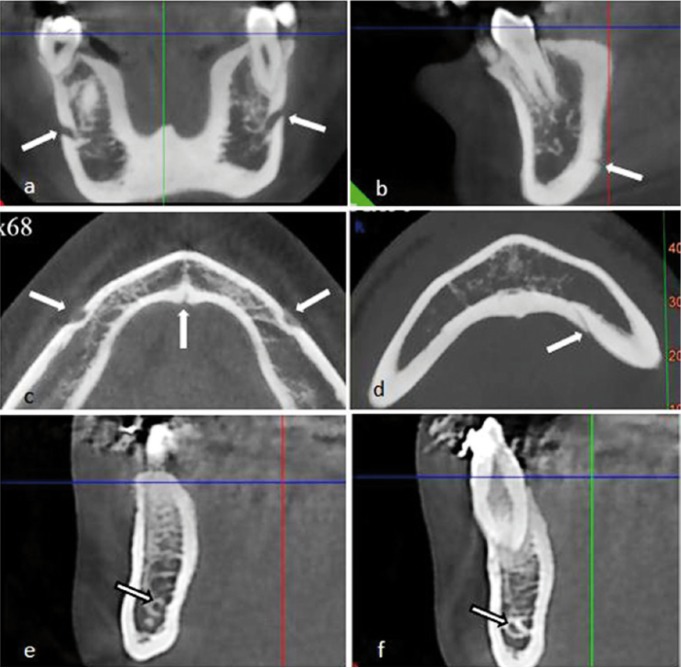
Coronal reformatted cross-sectional image showing mental foramina of a 17-year old boy (a), lingual foramen (b). Axial view of a 14-year old a boy show inf mental and lingual foramina (c,d). Incisive canal (on right and left side of the interforaminal region) shown on reformatted cross-sectional images as a rounded radiolucent area surrounded by a radiopaque rim representing the canal walls (arrows) (e,f).

All images were scored by two well-trained dental specialists. Evaluated landmarks are denoted in figures 1,2.

In the next step, selected cases were independently reevaluated by the two examiners to diagnose and classify the cases into different abnormality subtypes, such as congenital changes, malignant and benign tumors, odontogenic lesions, bone-related lesions, traumatic lesions (bony fractures), and inflammatory lesions (mucosal thickening, retention cysts, opacification, sinus polyps, and antroliths). Data were gathered and divergences between the examiners were solved by reaching a consensus.

-Statistical analyses

All calculations were processed using the Statistical Package for Social Science statistical software (version 16; SPSS Inc., Chicago, Illinois). Descriptive statistics including tables were used to display information. A chi-square test was used to compare the CBCT and panoramic images. Kappa statistics were also used to assess inter-examiner consistency.

## Results

-Visibility rating of anatomical landmarks on panoramic radiographs

**Figure 3:**
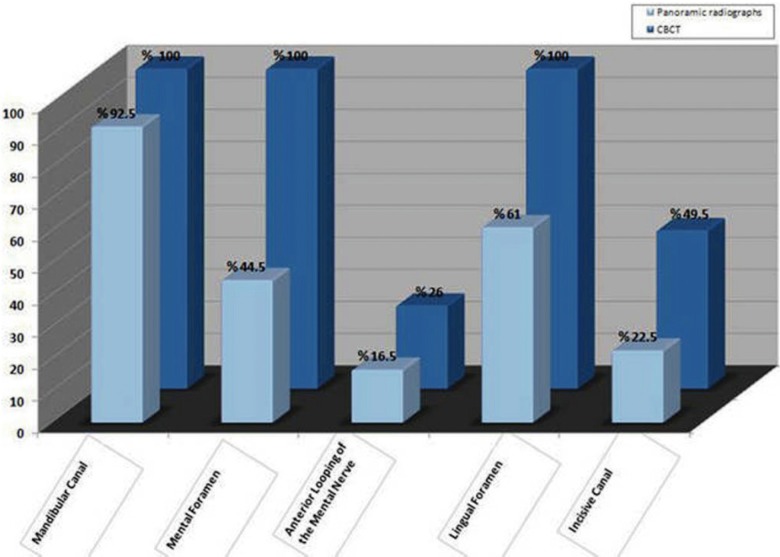
Good visibility of anatomical structures in each group.

**Figure 4:**
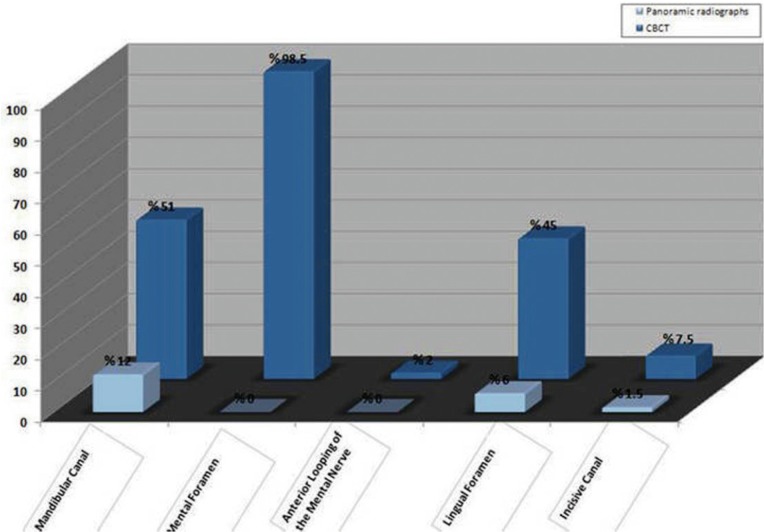
Appearance of anatomical structures in each group.

The visibility rating score in percentage of different anatomical landmarks, as illustrated on panoramic images, is shown in figures 3,4. The mandibular canal could be observed in 92.5% of 100 cases, with good visibility in 12.0%. The mental foramen could be observed in 44.5% of cases, while no cases had good visibility. Anterior looping of the mental nerve was present in 16.5% of cases, but no cases had good visibility. An incisive canal could be identified in 22.5% of cases, with only 1.5% showing good visibility. The lingual foramen could be visualized in 61.0% of cases, with good visibility in only 6%.

-Visibility rating of anatomical landmarks on CBCT scan images

The visibility of anatomical landmarks on CBCT scan images is shown in figures 3,4. The mandibular canal, the mental foramen, and the lingual foramen could be observed in 100% of cases, with good visibility in 51.0%, 98.5%, and 45.0% of cases, respectively. Anterior looping of the mental nerve was present in 26% of cases, with 2% having good visibility. An incisive canal could be identified in 49.5% of the cases, with only 7.5% showing good visibility.

Kappa statistics indicated excellent agreement for the observations of the anatomical landmarks as compared to the expert consensus statement. Kappa values for the panoramic images were 0.94, 0.99, 0.89, 0.90, and 0.86 for the mandibular canal, mental foramen, incisive canal, lingual foramen, and anterior looping, respectively. Additionally, Kappa values for the panoramic images were 1.00, 1.00, 0.93, 1.00, and 0.96 for the mandibular canal, mental foramen, incisive canal, lingual foramen, and anterior looping, respectively.

-Other findings

 Table 2 represents all of the findings from the 100 scans.

**Table 2:**
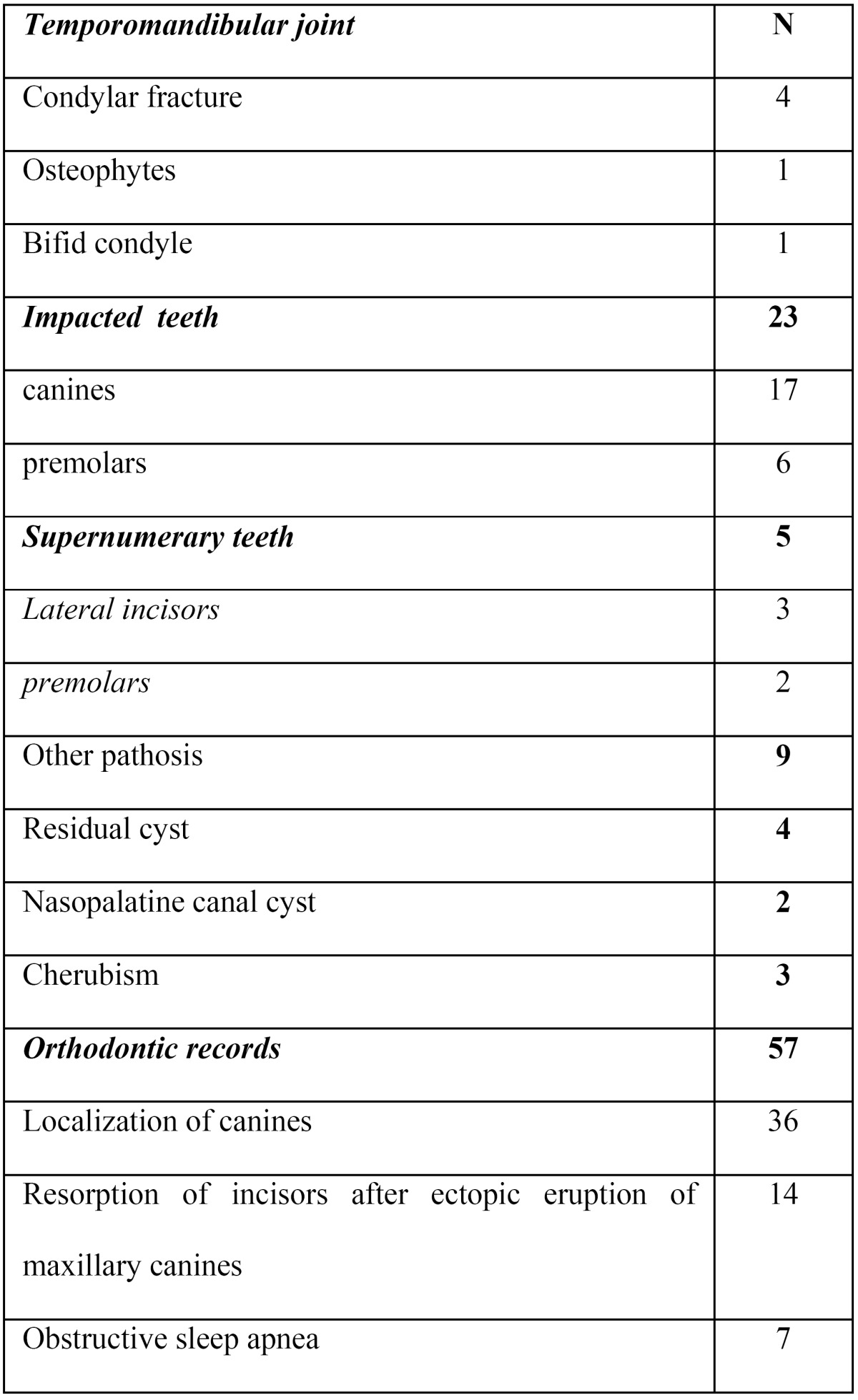
Summary of all the findings seen in the 100 CBCT scans (57 boys and 43 girls).

## Discussion

Several factors contribute to the reliability of landmark identification in children: the density and sharpness of images, the anatomic complexity and superimposition of hard and soft tissues, the definition of the landmark, and the training level or experience of the observers, especially for pediatric dentistry (10,11).

The mandibular canal could be observed on panoramic and CT scans in 92.5% and 100% of cases, with good visibility in 12.0% and 50% of them, respectively. These findings are less promising than those from a previous study on panoramic images, where a canal was visible in 99% of cases and good visibility in 49%; (2) however, in accordance with prior studies on CT scans, a canal was visible in 97% and 99% of cases in present study (3,12).

Olivier (13) was the first to describe the course of the incisive nerve as a continuation of the inferior alveolar nerve traveling through a canal. Mardinger et al. (6) and Mwaiva et al. (7) anatomically observed an incisive canal in 80% and 96% of mandibles, respectively. Other studies, however, have neglected to identify the presence of a true incisive canal. (14,15) The present anatomical study did not fully confirm the existence of the incisive canal, as it could only be seen in 49.5% (CT scan) and 22.5% (panoramic images) of the mandibles. The relatively low occurrence rate of the incisive canal in anatomical studies makes this observation most likely an anatomical variation.

In this study, mental foramina could be identified in 44.5% (panoramic images) and 100% (CT scan) of cases, which is lower the findings of other studies on panoramic images (3,12) and in accordance with CT scan studies (2).

An important anatomical variation in the interforaminal region is the anterior looping of the mental nerve that was present in 26% (CT scan) and 16.5% (panoramic images) of cases. This is in accordance with the results of Misch and Crawford (16) and Jacobs et al., (2) who reported radiographic visibility of anterior looping on panoramic images in 12% and 11% of cases, respectively. On panoramic radiographs, Ngeouw et al. (17) observed anterior looping in 40.2% of lower jaws. The value reported in the CT scan analysis done by Jacobs et al. (3) is somewhat lower (7% of the cases). Generally, the radiographic visibility may differ, to some extent, from the anatomical observations. However, Kaya et al. (18) and Uchida et al. (19) reported prevalence rates of 34% and 71% of anterior looping using CT images, respectively. Bavitz et al. (20) noted anterior looping in 21% of the cadaver mandibles they investigated. Variations in the reported incidence of this anatomical variant may depend on the criteria used to define anterior looping and the degree of resorption of the investigated mandibles.

The visibility of the lingual foramen using various conventional radiographic techniques has been documented in several radiographic studies, (2,4,6,7,21) in which the limitation of plain film and panoramic radiography in identifying these structures is documented. The results of this observational study demonstrate that the lingual foramen is visible in 61.0% (panoramic image) and 100% (CT scan) of cases. These findings are upper with results reported by several authors (3,8,22), who found that the lin-gual foramen was visible in 82% to 89% of cases when using a CT scan. On the other hand, our lingual foramen imaging results from panoramic images are significantly weaker than those of Jacobs in which the lingual foramen was visible in 71% of cases using panoramic images (2). When comparing results of the present study to those of other studies, ours are inferior to those re-ported anatomically (23,24) for a number of reasons, apart from image quality of the CBCT and panoramic equipment, including patient examination procedure limitations (e.g., subtle patient movement), degradation of the image quality due to soft tissue scattering radiation, the degree of corticalization of the canal wall, or combination of the above.

CBCT in dentistry has provided an imaging solution that has none of the projection errors associated with magnification and none of the superimposition problems associated with traditional panoramic imaging (25). In addition, CBCT has a wide range of tools such as 3D reconstructions in any direction to permit accurate identification of landmarks. Studies have reported excellent accuracy of 3D computed tomography (CT)(26,27). In our study, both panoramic (2D) and CBCT (3D) were used, the identification of landmarks reflected a real clinical situation, and discrepancies in landmark identification were likely.

CBCT has probably been one of the most revolutionary innovations in the field of dentistry in the past decade, and it provides a novel platform for imaging of maxillofacial area (28). It also provides clear and accurate images of structures, and therefore is extremely useful for assessing the bone component. As the resultant images displayed are often corrected for magnification, accurate measurements can be derived from the reformatted 3D data (29). Radiographs in 2D are insufficient, especially in complex cases like impacted teeth, supernumerary teeth, and orthognathic surgeries. CBCT images provide far more detailed information than conventional 2D radiographs and are user friendly. Soft tissues, the skull, the airway, and dentition can be observed and measured on CBCT images at a 1:1 ratio. In terms of its clinical significance, CBCT provides an excellent tool for accurate diagnosis, more predictable treatment planning, more efficient patient management and education, improved treatment outcome, and patient satisfaction.

The radiation doses from CBCT are significantly lower than medical CT, but generally higher than conventional dental radiography (30). Recently, the Sedentexct working group proposed provisional evidence-based selection criteria with clinical indications as to when CBCT should be performed (30). CBCT should only be used when the clinical question cannot be answered by conventional radiography, and the field of view (FOV) should be limited to the region of interest (31). Ideally, CBCT equipment should be able to offer a choice of volume sizes to reduce patients’ radiation exposure levels. A risk-benefit analysis must be performed on each individual patient when CBCT is being considered. In order to assess the risk of CBCT, the effective dose must first be calculated as well.
